# Early user perspectives on using computer-aided detection software for interpreting chest X-ray images to enhance access and quality of care for persons with tuberculosis

**DOI:** 10.1186/s44263-023-00033-2

**Published:** 2023-12-21

**Authors:** Jacob Creswell, Luan Nguyen Quang Vo, Zhi Zhen Qin, Monde Muyoyeta, Marco Tovar, Emily Beth Wong, Shahriar Ahmed, Shibu Vijayan, Stephen John, Rabia Maniar, Toufiq Rahman, Peter MacPherson, Sayera Banu, Andrew James Codlin

**Affiliations:** 1 Stop TB Partnership, Geneva, Switzerland; 2 Friends for International TB Relief (FIT), Hanoi, Vietnam; 3 Department of Global Health, WHO Collaboration Centre On Tuberculosis and Social Medicine, Karolinska Institutet, Stockholm, Sweden; 4 Centre for Infectious Disease Research in Zambia, Lusaka, Zambia; 5 Socios En Salud Sucursal Peru, Lima, Peru; 6 Africa Health Research Institute, KwaZulu-Natal, South Africa; 7 Division of Infectious Diseases, Heersink School of Medicine, University of Alabama Birmingham, Birmingham, AL USA; 8 International Centre for Diarrhoeal Disease Research, Bangladesh (icddr,b), Dhaka, Bangladesh; 9 PATH India, Mumbai, India; 10 Janna Health Foundation, Yola, Nigeria; 11 Interactive Research and Development (IRD) Pakistan, Karachi, Pakistan; 12 School of Health & Wellbeing, University of Glasgow, Glasgow, UK; 13 Malawi-Liverpool-Wellcome Trust Clinical Research Programme, Blantyre, Malawi; 14 London School of Hygiene & Tropical Medicine, London, UK

**Keywords:** AI, Tuberculosis, Screening, Use cases, Thresholds, Implementation

## Abstract

Despite 30 years as a public health emergency, tuberculosis (TB) remains one of the world’s deadliest diseases. Most deaths are among persons with TB who are not reached with diagnosis and treatment. Thus, timely screening and accurate detection of TB, particularly using sensitive tools such as chest radiography, is crucial for reducing the global burden of this disease. However, lack of qualified human resources represents a common limiting factor in many high TB-burden countries. Artificial intelligence (AI) has emerged as a powerful complement in many facets of life, including for the interpretation of chest X-ray images. However, while AI may serve as a viable alternative to human radiographers and radiologists, there is a high likelihood that those suffering from TB will not reap the benefits of this technological advance without appropriate, clinically effective use and cost-conscious deployment. The World Health Organization recommended the use of AI for TB screening in 2021, and early adopters of the technology have been using the technology in many ways. In this manuscript, we present a compilation of early user experiences from nine high TB-burden countries focused on practical considerations and best practices related to deployment, threshold and use case selection, and scale-up. While we offer technical and operational guidance on the use of AI for interpreting chest X-ray images for TB detection, our aim remains to maximize the benefit that programs, implementers, and ultimately TB-affected individuals can derive from this innovative technology.

**Supplementary Information:**

The online version contains supplementary material available at 10.1186/s44263-023-00033-2.

## Background

With advances in computing power, data availability, and theoretical understanding, artificial intelligence (AI) has undergone an evolutionary expansion. Advancing from basic applications, such as optical character recognition, applications using AI have become an essential part of industry, military, government, and leisure. Whether through speech and face recognition, automated tagging of friends on social media, or receiving new music recommendations, AI has deeply penetrated the fabric of society and our everyday lives with the rise of large language models such as ChatGPT epitomizing the latest chapter of this trend [1, 2]. AI in the healthcare field has also experienced a similar exponential growth. The technology is routinely used in disease screening for cancer [3], trauma [4], endoscopy [5], and other conditions [6] to interpret medical images.

More than 10 million people develop tuberculosis (TB) each year, and an estimated three million are missed by national TB programs (NTP) [7]. While microbiological tests are used to diagnose TB, chest X-ray (CXR) has long been employed for screening and assisting diagnosis, given its high sensitivity and cost-effectiveness. Prevalence surveys conducted in 33 high TB-burden African and Asian countries showed that 30–79% of people with microbiologically confirmed TB did not report TB symptoms and were detected only by CXR [8, 9]. Computer-aided detection (CAD) software for interpreting CXRs during TB screening emerged around 2010 when several studies documented promising performance [10, 11]. However, neither the End TB Strategy nor its digital health agenda published in 2015 addressed the inclusion of CAD software in TB programs [12, 13].

In 2016, the World Health Organization (WHO) considered developing guidelines on CAD software for TB, but concluded there was insufficient evidence. At that time, only one CAD software solution was commercially available, and there was disagreement about standards for software performance in contrast to the clear diagnostic gold standard of microbiological testing [14]. In the following years, the CAD software for TB landscape and evidence base grew exponentially [15, 16]. At least 17 CAD solutions for TB have obtained a *conformité européenne* (CE) mark [17]. Initially, evaluations of CAD software for TB compared the accuracy and performance of software relative to human readers for detecting pulmonary abnormalities [18–20]. These studies were followed by evaluations and systematic reviews comparing CAD software solutions systems against one another [21–24].

In 2021, these trends culminated in the WHO revising its TB screening guidelines to recommend CAD software as an alternative to human readers for interpreting digital CXRs to screen and triage individuals aged 15 years and older for TB [14]. This milestone is noteworthy as it is the first time the WHO has issued guidelines on the use of AI software in any disease field [14]. As the literature on CAD software for TB continues to grow, so does demand; the focus of implementers is now shifting from “whether to use” to “how to implement” this technology. Unlike recently published recommendations [25, 26], the WHO-revised TB screening guidelines did not include an operational handbook for program implementers to guide new implementers.

**Fig. 1:**
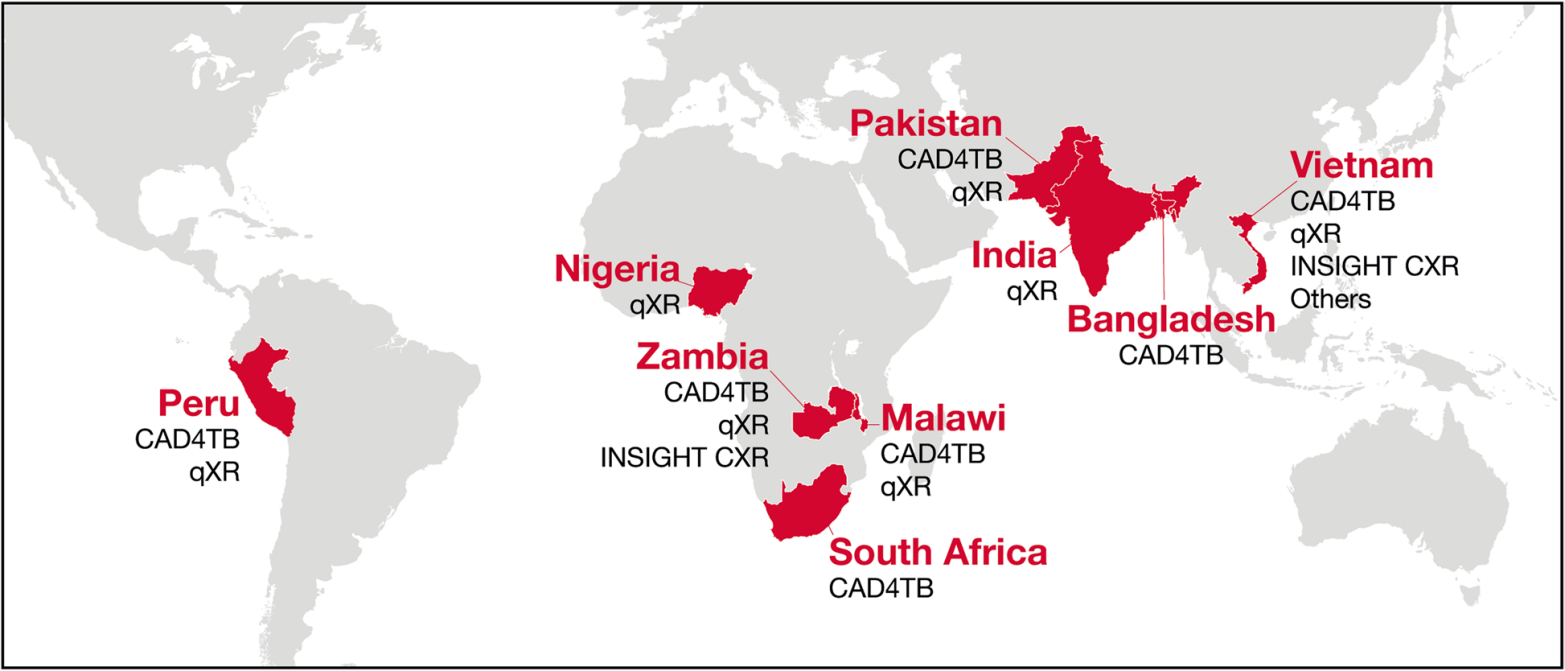
Geographies and CAD software platforms CAD4TB (Delft Imaging, The Netherlands); INSIGHT CXR (Lunit, South Korea); qXR (Qure.ai, India); DrAid (Vinbrain, Viet Nam)

Despite growing numbers of publications, accounts from implementers based on prospective CAD software deployments during TB screening remain limited [27, 28]. Here, we document field experiences in implementing and supporting CAD software deployments in various high TB-burden settings. The primary purpose of the CAD was for pre-diagnostic screening, but may also include diagnostic support functions on clinical studies, prevalence surveys, active case finding (ACF), and expansion of TB Preventive Therapy (Fig. 1 and Additional file 1: Table S1). Most of the perspectives presented are based on three CAD software solutions that were included in the WHO TB screening guidelines: CAD4TB (Delft Imaging, The Netherlands), INSIGHT CXR (Lunit, South Korea), and qXR (Qure.ai, India). However, we endeavored to generalize our experiences to guide users and developers on key considerations when deploying any CAD software for TB screening. Although our aim is to offer technical and operational guidance, it is important to maintain focus on the outcome of this work, which is to maximize the benefit that programs, implementers, and ultimately TB-affected individuals can derive from this innovative technology.

### Overview of CAD software for TB screening

The WHO End TB Strategy highlights systematic screening and early detection as critical interventions to achieve global targets in TB incidence and prevalence reduction [29]. An important tool for screening persons with signs of TB and triaging those unlikely to benefit from further evaluation is CXR. It can also be useful for clinical diagnosis when it is not possible to confirm TB disease bacteriologically. However, when deploying CXR for TB screening, one of the key considerations is the availability of skilled personnel to conduct CXR examinations, maintain the equipment, and read the results [14]. Particularly with respect to the latter, consensus is growing that CAD can play a crucial role in the reduction of the global TB burden and improvement in public health around the world [16, 30].

The use of CAD software for CXR interpretation requires a digital CXR image, either computed or direct digital radiography, or the digitization of analog films. The software processes CXR images as a Digital Imaging and Communications in Medicine (DICOM) file, although some software can also handle other file types (e.g., JPEG files), which, albeit untested, could expand CAD access to analog CXR systems that remain the current standard of care in many low- and middle-income countries (LMIC). Most CAD software solutions operate on a cloud-based server requiring a stable internet connection with a growing array of locally hosted, offline processing options.

Depending on the specific CAD software, various outputs are generated after CXR image processing. Most often this includes a probabilistic abnormality score (e.g., 1–100 or 0.01–1.00), with higher scores indicating a greater likelihood that the abnormalities in the CXR image are caused by TB. This continuous abnormality score can be dichotomized at a selected threshold, above which individuals are indicated for a follow-on TB evaluation. Threshold selection therefore represents a critical choice between prioritizing technical and allocative efficiency of follow-on evaluations and the likelihood and consequences of missing people with “true positive” TB disease. In the absence of clear guidance and standards, certain CAD software solutions operate with a default threshold score which can be updated in collaboration with the manufacturer, while others leave threshold selection entirely to the implementer’s discretion. Some studies have shown excellent performance with manufacturer preset thresholds like qXR from Qure.ai for example, while others have shown additional calibration could improve performance depending on intervention goals [31, 32]. CAD software solutions also often produce a heat or segmentation map highlighting areas of abnormality in the CXR image, and increasingly, they are also providing other annotated findings in addition to a TB score, which can be used as a CAD-generated radiology report (e.g., consolidation, fibrosis, nodules and other opacities, cavity, blunted costophrenic angle, pleural effusion, hilar prominence, and cardiomegaly).

### Threshold score selection and use

The selection of a threshold score influences the ultimate sensitivity and specificity of a CAD software’s CXR interpretation. This selection could mean the difference between being detected or unreached for TB-affected persons, meeting or missing public health targets, and achieving allocative efficiency or wasting precious public health resources. Unlike with human readers, a threshold score can be lowered to be more sensitive and less specific, or raised to be more specific and less sensitive for each captured image. However, no threshold selection point is likely to ever be 100% sensitive or specific, and the best threshold represents an optimization of interpretation accuracy and follow-on workloads for your CAD software use case, as discussed in the next section. While the concept of threshold selection may seem intuitive, it remains contentious among policymakers and can be challenging for new implementers.

In the absence of clear guidance, strategies for threshold selection were diverse and unstructured. While early studies from Zambia and Pakistan used pre-defined scores, they employed different software versions limiting comparability and generalizability [10, 22, 33]. Implementers in South Africa and Bangladesh found varying abnormality score distribution and ideal thresholds across settings, AI software, and versions of the same software [19, 34, 35]. To assist implementers, the WHO’s Tropical Disease Research (TDR) unit has published a process to select setting-specific thresholds with empiric evidence [36]. This involves conducting screening procedures as planned, but not using CXR results to guide clinical decisions until a well-characterized dataset with sufficient TB-confirmed images can enable meaningful inferences about CAD software performance. Such studies tend to be impractical from time and cost perspectives, can delay prospective use of the software, and would need to be regularly repeated as the optimal threshold selection changes in the screened population and the software type and version.

Alternatively, if available, CAD software could be deployed in parallel to human readers for a short duration, where sputum is collected for any abnormal CXR interpretation. For example, this includes instances where the radiologist and CAD software have discordant results, but, in many settings, this is not an option. If a screening cohort classified as abnormal by the human reader and as normal by the CAD software does not yield persons detected with TB, then the CAD software’s threshold is achieving equivalent sensitivity with the human counterparts. Experienced implementers resorted to this method of real-time in-house data analysis for continuous threshold optimization for the sake of expediency, especially in the early days and often in close collaboration with the CAD software developers [27]. The concurrent, real-time refinement of the threshold score, potentially supplemented by clinical evidence, using these alternative methods, was particularly important for large-scale ACF programs, which experience large heterogeneity in CXR throughputs, follow-on testing capacities, and target populations. This concept has now been formally described as iterative operating point calibration (ITSC) and is currently undergoing pilot testing. This mode enables TB programs to select and iteratively improve a threshold score using data gathered during implementation until desired programmatic targets are reached using a systematic approach [37].

**Fig. 2:**
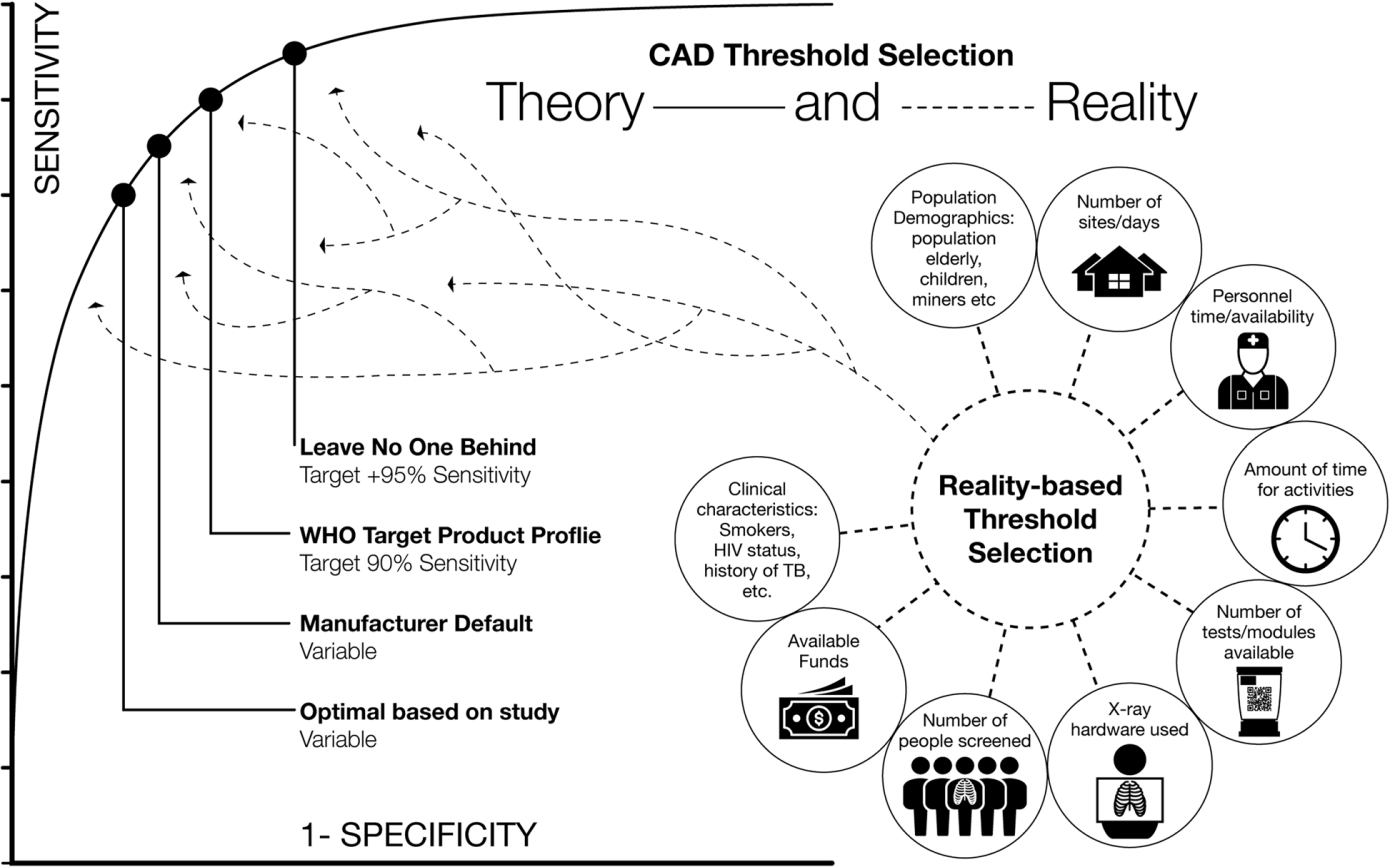
Theory and reality of threshold selection

While threshold selection studies and parallel radiologist/CAD reading can provide important data on the most efficient settings and even produce universal threshold scores for implementers to rely upon, it remains critical to retain the flexibility to match real-world exigencies of TB screening [38]. Specifically, implementers have praised the ability to raise threshold scores and match locally available diagnostic testing capacities and workforce, and to lower threshold scores and increase detection during the same screening campaign in different settings (Fig. 2). This flexibility is a key strength of the CAD software, compared to human readers who in some settings have found it challenging to adjust professional opinions and habits. However, this versatility also highlights another critical point. While the issue of threshold selection will likely continue to be an area of focus at global and implementation levels, implementers emphasized the importance of not losing sight of other priorities that would benefit from but have not received a similar level of attention and debate. Some of these considerations have been further explored below.

### CAD software use cases

**Fig. 3:**
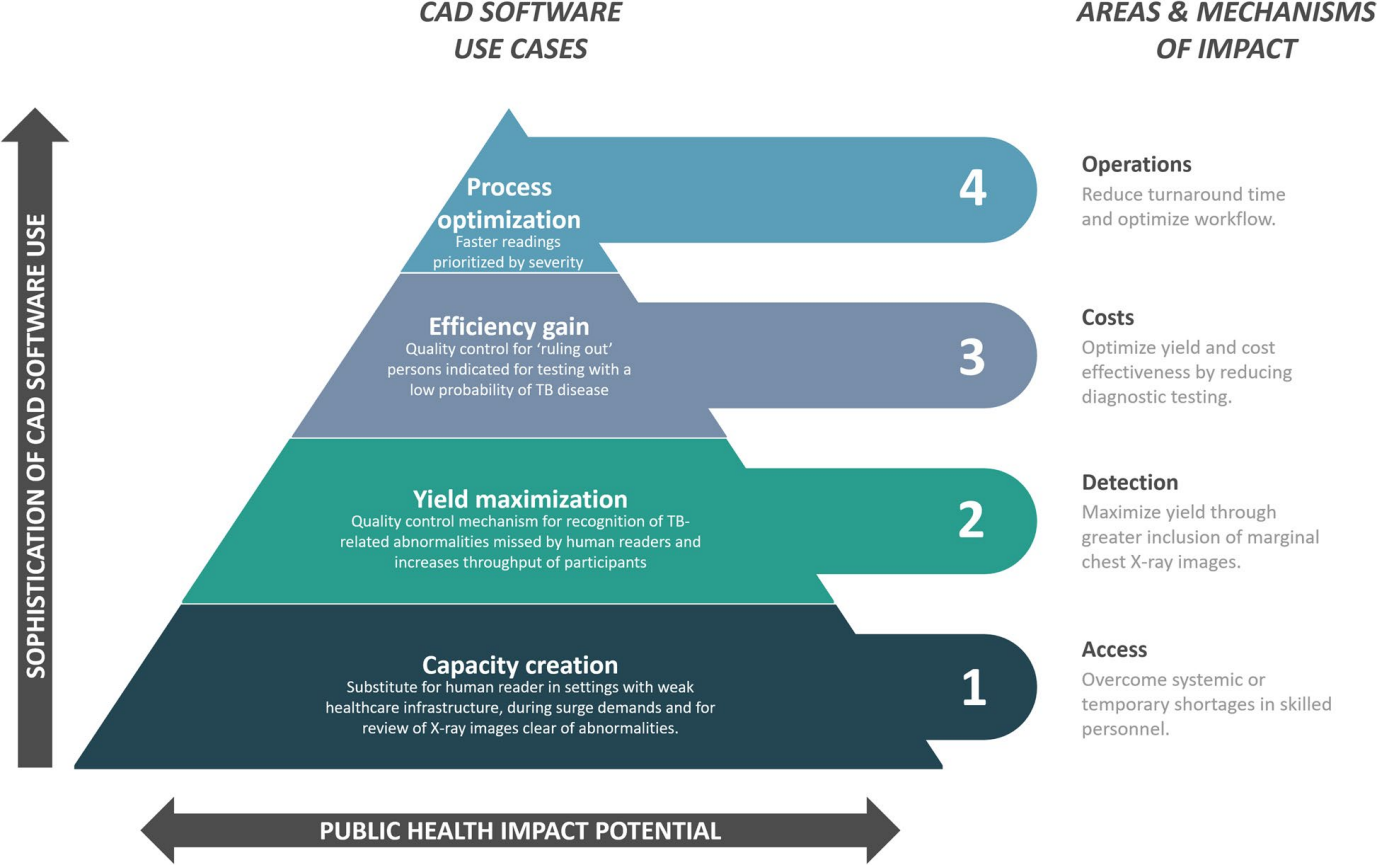
Summary of CAD software use cases and potential public health impact

The use case is a key criterion to determine the CAD software’s utility and autonomy for users and the potential public health impact they seek to achieve. From our user accounts, we synthesized four major CAD software use cases in ascending order of sophistication and potential public health impact: capacity creation, yield maximization, efficiency gain, and process optimization (Fig. 3). While aspects of each use case may overlap, they represent different approaches to improve TB care quality and processes when designing programs.

The *capacity creation* use case aims to overcome access barriers related to CXR interpretation. These barriers result from a systemic lack of skilled personnel. For instance, in Adamawa State, Nigeria, there were only two certified radiologists in the public sector in 2022 to serve an estimated population of 4.5 million. Shortages can also be caused by many factors, including remote geographies and low population density, lack of supporting healthcare infrastructure, low background prevalence where TB is not considered a public health issue, or surge demands, such as diversion of health staff for health emergencies or waves of ACF [39–41]. In these situations, CAD software can be a valuable substitute for human readers [42, 43]. Another form of capacity creation is through allocative efficiency of human reader time. This may be achieved by first processing CXR images with CAD software to identify and triage ones that are clear of abnormalities away from human reading. This CAD software use case can substantially reduce a human reader’s workload [44] to focus on thorough examination of abnormal images, patient consultations, and other value-added tasks [43]. This can ultimately raise the quality of care provided to the patient.

CAD software can also achieve substantial *yield maximization* in situations where humans represent the primary CXR readers. Specifically, a CAD software can improve TB screening yields by identifying abnormalities that human readers are unable to recognize [45] or unwilling to interpret as TB-related, resulting in more at-risk people being eligible for diagnostic testing, as is currently the standard of care of one implementer in Bangladesh. Missing TB-related abnormalities apply particularly to general care settings, where TB is not the primary focus of service delivery. In these settings, CAD software can also fulfill a passive surveillance function to alert clinicians to consider TB during interpretation or differential diagnosis [46]. Failure to recognize TB due to low awareness has been noted as a factor associated with diagnostic delay [47], and using CAD software acting as a passive background screen for TB in high-volume hospitals can improve TB detection as an implementer in India recorded a yield increase from 18 to 27% at nine peripheral hospitals in Mumbai (personal communication from SV). In addition, CAD software-enabled CXR screening programs may be able to screen more people in a day, as CAD software does not suffer from reader fatigue. In certain settings, particularly those with limited access to health services, CAD software may be viewed as cutting-edge technology that they could not otherwise access, leading to higher participation among target populations.

The *efficiency gain* use case is to “rule out” people already indicated for diagnostic testing, for example, after a positive verbal screen for TB symptoms. People with TB symptoms are further screened by CXR to optimize screening yield and cost-effectiveness by reducing diagnostic testing after a “false positive” symptom screen but normal CXR. This use case leverages the high negative predictive value of CXR to rule out people with low likelihood of TB with minimal yield loss [48]. Similar to the prior use case, CAD software can aid less experienced radiographers and clinicians in the event that a CXR exhibits parenchymal abnormalities that are not clearly discernable [49]. For example, at the height of the COVID-19 pandemic in Vietnam, TB screening without the use of a CAD software was piloted at a COVID-19 quarantine center as a measure to ensure continuity of TB care (personal communication from LNQV). Of the 365 COVID-19-infected individuals, 110 were indicated for Xpert testing, but none tested positive for or were clinically diagnosed with TB. A well-trained CAD software may have helped to avoid the unnecessary testing costs but evidence is lacking. In response, a number of developmental and commercial solutions have upgraded their algorithm to integrate screening for both TB and COVID-19 [50–53], as well as other respiratory conditions [54, 55]. Continuous developments for pneumonia, heart disease, chronic obstructive pulmonary disease (COPD), asthma, and lung cancer are other areas of ongoing CAD software development [56–62]. This means that TB can be mainstreamed as part of a holistic, CAD-assisted respiratory care package. As highlighted by accounts from Malawi, the synergies of assessing one CXR for a broad range of respiratory ailments represent a clear efficiency gain for health systems and patients, while helping LMICs manage the rising double burden of communicable and non-communicable diseases [63, 64].

Lastly, CAD software solutions can be useful for *process optimization*. One example is the reduction in turnaround time (TAT) for a CXR result [65]. In India, TAT was reduced by 41% thanks to CAD-supported CXR interpretation [66]. Worklist prioritization and severity assessments represent another form of TAT optimization [67]. Simulations of clinical workflow have shown that AI can reduce report TAT for critical findings. Instead of using the standard First-In-First-Out workflow, AI can prioritize more critical cases for urgent care [68, 69]. This use case also benefits high-volume CXR settings and interventions such as respiratory departments in quaternary general hospitals, perhaps even in multiple simultaneous sites such as during national TB prevalence surveys. In these settings, access to skilled radiologists, although available to some extent, can be quickly depleted as image volumes increase [34]. Precise adherence of human readers to guidelines on CXR interpretation can thus be challenging and corresponding intra- and inter-reader variability is well documented [11, 70, 71]. AI provides results that are consistent, adaptable without retraining, and are unaffected by fatigue, stress, or lapses of concentration.

### Deployment

Deployment of a CAD software is primarily a digital health intervention. Therefore, budgeting for IT, human resources, and data management support to install and maintain the software, as well as linking and operating multiple databases, is critical. Choosing a cloud-based or offline deployment forms another important deployment decision. Most CAD software providers prefer to operate in the cloud as this deployment modality is cheaper, quicker to troubleshoot, and easier to upgrade to new services and software versions. In addition, the ability to synchronize data in real time, perhaps to obtain a second opinion of the CXR and CAD results for complicated cases via an access by a central expert radiologist, for instance, can aid in quality control. However, cloud solutions are dependent on stable internet connections with high upload bandwidth, which may exclude this deployment modality in many areas (e.g., remote areas in Nigeria or prisons in Peru). Even when high upload bandwidth connections are available, the size of each individual DICOM file and the frequency with which they are created during TB screening can strain connections. In settings with metered connections, users must ensure that there is enough credit to support significant data transfers (sometimes larger than 5 GB of data, depending on the size of the DICOM files and number of participants screened). CAD software downtime can cause major delays and disturbances in screening practices, particularly when they are not used in conjunction with an on-site human reader. Nevertheless, Pakistan has conducted more than 2 million CXR screens using CAD software through networked mobile units, highlighting that well-placed and coordinated cloud solutions are possible at scale.

Compared to cloud systems, self-contained offline systems are unburdened by these challenges. However, they require additional costs for physical hardware and accessories. Offline deployments usually require expensive laptops with high-end computing and graphical processing power. Nevertheless, the box solution that locally processes the DICOM images has lower processing power than an online server. If DICOM files are captured too quickly, an offline box solution can become overwhelmed and backlogged, indicating that offline CAD solutions often need to be deployed in a specific fashion which optimizes the throughput of participants. Meanwhile, their installation and maintenance can be cumbersome. For example, laptops included in X-ray systems are often locked for third-party software, requiring a secondary system for CAD deployment, although more CAD software providers are working with X-ray vendors to address this, especially with portable devices. For example, implementers in the Philippines recently were able to deploy Fujifilm’s Xair system with Delft’s AI, and Fujifilm has integrated solutions with other providers. A new promotional strategy has also included bundling these portable systems with an in-house or white-label CAD software for a comprehensive screening package. Often, the biggest hurdle in setup is the integration of the CAD software with the third-party image acquisition software different from the X-ray systems used. In Nigeria, a pre-integrated solution between Qure.ai and MinXray provided simplified completely wireless use in remote areas with no electricity, and Qure.ai has also partnered with LTE Medical Solutions in South Africa [72]. A supplier in India, Mylab Discovery Solutions, is in talks with several AI providers for their handheld X-ray device, confirming the trend towards these comprehensive solutions for TB screening deep in affected communities [73, 74].

Deployment of CAD software solutions with existing facility-based machines may be more complicated. This complexity can be a result of a litany of factors. Some implementers experienced incompatibility between the CAD4TB software and non-Delft X-ray systems. Multiple X-ray manufacturers will use different versions of image acquisition software, which complicates pushing images for AI reading. Deployment of qXR boxes at several hospitals in Vietnam encountered challenges due to Picture Archiving and Communications System (PACS) server incompatibility, and ultimately required manual DICOM file transfer, as well as network security-related complexities. Early CAD4TB versions had problems reading 16-bit DICOM files, necessitating the wait for a manufacturer upgrade [75].

Mature CAD software solutions generally offer intuitive, user-friendly interfaces. These provide a backend database to capture patient information, lab results, as well as a CXR viewing platform with multiple login offerings for remote access. At large scale and as part of programmatic deployment, integrated data management software will be a critical component of any CAD software for CXR screening solution. Some implementers were able to leverage these systems as temporary substitute for electronic medical record management, especially when operating in remote areas with limited health infrastructure. The integration of CAD with the broader healthcare system and the establishment of interoperability among national health information systems are crucial next steps. The inherent digital nature of CAD software tools offers a promising chance to align digital ecosystems for TB surveillance. Many CAD software providers offer an application programming interface that enables seamless connection between X-ray and CAD outcomes and the designated data system of end-users. The current challenges in most high TB-burden countries primarily stem from the absence of a nationally standardized unique citizen identifier, inconsistent data structures in comparison to legacy systems, and inadequate IT infrastructure, particularly in peripheral settings.

Several implementers did not use medical personnel as part of the screening process (i.e., recruitment, image capture, sputum collection). In other settings, such as Vietnam and Bangladesh, local regulations require physicians or radiographers to be present and available during CXR operation. The user experience is further enhanced by the simplicity of threshold scores to delegate decisions on sputum collection to project staff and even lay workers. However, this level of decentralization, while a core value proposition of these CAD software solutions, is highly dependent on the local regulatory context and the risk appetite of the implementer in their reliance on software not approved for clinical decision-making by a Ministry of Health. As the use of AI in healthcare expands, it will put additional pressure on local regulators to adapt.

A final deployment consideration relates to equity of access and utility for persons affected by TB. It is well understood that active screening for TB and extending access to innovative tools promotes equity by extending high-quality care to people facing structural, geographical, and financial barriers [76]. Conversely, it has been postulated that health equity could be adversely affected by new technologies such as CAD. For example, more accurate versions are not made available equitably to all users [77]. In addition, versions with suboptimal performance could be introduced too rapidly without rigorous evaluation due to scarcity of high-quality alternatives. Thus, a rights-based approach to deployment that focuses on the provision of person-centered care and the best quality health services based on individual needs rather than public health burdens and budgets constitutes an additional critical deployment consideration [78].

### Barriers to scale-up

Globally, an estimated 3–4 billion CXRs are performed every year [42, 79]. In LMIC settings, many of these CXRs rely on the current paradigm of facility-based, analog CXR systems. Hence, additional investments in X-ray digitization and portability are unavoidable for CAD software uptake to expand. In addition, lack of data on the impact of CAD software utilization on clinical outcomes may also inhibit uptake [80]. However, this technology also faces key systemic barriers to scale-up that are unlikely to be solved through more funding or stronger evidence alone. These barriers principally are regulatory, economic, and transactional in nature.

In terms of the *regulatory barriers*, convincing policymakers to permit the integration of AI software into health services may pose a considerable challenge due to the associated liabilities. One key liability is the scientific and medical basis on which to base clinical decisions. Despite the publication of international guidance, in many high TB-burden settings, government bodies may require locally obtained evidence prior to translating WHO recommendations into national policy. Another liability surrounding quality assurance through certification has been discussed elsewhere [81]. Liabilities may also encompass national information security and personal privacy. Personal identifiable information or at minimum DICOM files are often transferred, read, and stored in servers situated outside of the country of data generation. This puts these AI software providers outside of the jurisdiction of national authorities, raising questions about responsibilities and remedial actions in the event of a data or privacy breach. Regulatory barriers are exacerbated by regional, continually updated privacy laws such as General Data Protection Regulation (GDPR) in the European Union (EU) or the Health Insurance Portability and Accountability Act (HIPAA) in the USA. Even if these liabilities can be managed through diligent legal counsel and close coordination with competent authorities, regulatory risk mitigation requires resources and raises the cost of deployment to achieve and maintain compliance. However, some AI software developers have adjusted their practices to mitigate regulatory hurdles for their customers. EU-based Delft has standardized anonymization of DICOM files prior to storage, which is a practice that should become industry standard to also advance rights-based TB care.

*Economic barriers* exist despite the strong need for and utility of CAD software, as a result of the sole focus on TB. This focus thereby limits the addressable market and overall utility for the healthcare provider, particularly in mainstream facility-based settings. In these settings, a human reader is usually present, so that CAD software deployment aims to optimize rather than to create capacity. Thus, the marginal benefit-cost ratio is lower than in ACF settings, which in turn may suppress demand. One way that this barrier will be addressed is by expanding the market size through validation and greater uptake of the expanded CAD software capabilities for other diseases and health conditions mentioned above. Domestic funding may be more easily mobilized if CAD can be integrated to strengthen health systems through greater disease integration. A second barrier is that scaling up CAD software necessitates capital expenditure and raises operating costs to meet system requirements in digital X-ray equipment, PACS systems IT and communications infrastructure, and hospital management information systems. A tertiary cost factor is the availability of follow-on testing capacity and particularly molecular diagnostics. Nevertheless, as CAD software platforms continually improve, their marginal benefit increases, and global ambition for ending TB expands, adoption of CAD software is bound to grow as well. India and the Philippines are two key examples, where catalytic investments by The Global Fund and USAID were dedicated towards expanding CAD software for TB in both ACF and facility-based settings to overcome traditional economic barriers and shift the momentum towards scale-up of this technology [82–84].

*Transactional barriers* arise from uncertainties in CAD software pricing. Initial pricing models were set per-DICOM file processed, with a price point that often exceeds the abovementioned costs of a human reader in LMICs. Prices were volume-driven and negotiable, but transaction-based and opaque. However, the transactional pricing is giving way to more innovative, transparent, and buyer-centric pricing models based on licensing with unlimited reads. In late 2021, two CAD software products were added to the product catalogue of the Stop TB Partnership’s Global Drug Facility (GDF), a global pooled procurement mechanism. The Global Fund also has options through its various mechanisms [85], and a new GDF tender is underway to expand options for implementers. As the market matures, innovative models will emerge including tier-based licensing, freemium (i.e., free basic functions with premium options) or free/ad-supported schemes, as well as other hybrid pricing models commonly observed in Software-as-a-Service solutions to develop a competitive advantage in this rapidly expanding market. The selection of CAD and hardware vendors together is also a challenge for implementers. Agreements between different developers can lead to preferential pricing for bundled X-ray systems, and CAD software solutions may be another attractive selling proposition for greater uptake among end-users.

### Continuous evolution

Given the rapid developmental trajectory of CAD software solutions for TB [33, 70], they have been described as a moving health technology that will continuously evolve [36]. Current CAD software solutions do not use demographic and clinical data when calculating scores, even though studies have shown that humans and software perform differently in people with prior TB history, by age, and referral/screening site [17, 19, 21, 24, 86]. A human reader will often be able to consider additional demographic and clinical patient characteristics when reading an image for TB. This has been tested for lung cancer [87], and it behooves CAD software developers to upgrade solutions to develop and include this feature. Improved performance with additional data points also strengthens the argument for a local calibration effort prior to deployment to take into consideration regional differences in age distribution, history of TB, or smoking habits, for example. Particularly with respect to age distribution, a glaring evidence gap is the use of CAD software in children. It is noteworthy that despite the advantages of humans in this regard, CAD software performance for TB screening is on par with, or outperforms, human readers. With large datasets, it should be possible to develop more accurate individualized scores based on the characteristics of the individual screened, although this may have to be calculated by end-users.

CAD software that provide only a continuous abnormality score are not be ideal for programs that share CXR images and scores with clinicians. While some general practitioners liked the ability of the CAD software to show heat maps of potential abnormalities, others found it difficult to reconcile high abnormality scores with non-TB-related conditions, such as pneumothorax. Different CAD software solutions have different distribution in their abnormality scores, and high numeric scores without bacteriological confirmation of TB can cause both under- and over-diagnosis either due to reservations about the utility of the systems or due to an over-reliance on higher scores to diagnose TB clinically [24]. To compensate, it is becoming increasingly common for CAD software solutions and other machine learning approaches to produce radiology reports on specific lung conditions, which may have great potential to help healthcare workers with TB as well as other diseases [49, 88, 89]. However, further research is required to validate the accuracy and utility of these reports in clinical practice.

While global policy often trails the implementation of new tools, the explosive growth of AI in the healthcare setting necessitates a way to provide regular updates. Standard WHO guidance is expected to have a lifecycle of 3–5 years, but in the case of CAD software for TB screening, these lifecycles may have to be shortened. New software versions are released much more quickly than what would be expected of a diagnostic test or drug, and potential users should be able to easily understand the differences in performance and characteristics of successive versions of each CAD software. Similarly, new version upgrades would ideally be seamless without the need to re-calibrate thresholds, for example. However, some version updates make fundamental changes to the distribution of abnormality scores, like for example, CAD4TB v6 and v7, and recalibration is critical [90]. In these situations, communication from the developer is vital to avoid confusion [35]. There is an online resource for CAD software for CXR hosted by Stop TB Partnership and FIND, which is updated regularly [91], and TDR avails a number of digital tools to implementers [92]. However, a reliable, open-source evaluation dataset to benchmark CAD software performance and practical tools to help implementers optimize process flow integration of the CAD software remain urgently needed.

## Conclusions

There is little doubt that AI will continue to permeate our lives in general, and the same can be expected in global public health overall, and especially TB care and lung health. CAD software for CXR interpretation can address several current shortcomings for TB program implementers who are attempting to incorporate CXR screening to improve TB detection. These range from compensating for the lack of human resources, supporting high throughput ACF, and the ability to calibrate test performance based on specific settings and field conditions. The instantaneous generation of the results, and more recently, indistinguishable emulation of human radiology reports, are major value drivers of the new technology. However, there are many pre-requisites for the expanded use of this tool, including clear guidance on its role, use case, and thresholds, coupled with acceptance and support of implementers, as well as the appropriate healthcare and communications infrastructure. Ultimately, adoption will also heavily depend on the legal framework and greater acceptance of digital solutions in healthcare decision-making. The challenge will be for the ecosystem from legislator to end-user to keep pace to maximize the utility of this disruptive new tool for global public health purposes.

## Supplementary Information

**Additional file 1: Table S1.** Overview of Country Programs using Artificial Intelligence to Interpret Chest Radiographs.

## Data Availability

Not applicable.

## Abbreviations

ACF: Active case finding
AI: Artificial intelligence
CAD: Computer-aided detection
CE: Conformité Européenne
COPD: Chronic obstructive pulmonary disease
CXR: Chest X-ray
DICOM: Digital Imaging and Communications in Medicine
EU: European Union
GDF: Global Drug Facility
GDPR: General Data Protection Regulation
HIPAA: Health Insurance Portability and Accountability Act
ITSC: Iterative operating point calibration
LMIC: Low- or middle-income country
NTP: National TB Program
PACS: Picture Archiving and Communications System
TAT: Turnaround time
TB: Tuberculosis
TDR: Tropical Disease Research
WHO: World Health Organization
