# Supplementary material for: Early user perspectives on using computer-aided detection software for interpreting chest X-ray images to enhance access and quality of care for persons with tuberculosis
Source: BMC Glob Public Health. 2023 Dec 21;1:30. doi: 10.1186/s44263-023-00033-2 (PMC11622860; doi:10.1186/s44263-023-00033-2)
Supplement: Supplementary file 1 — Additional file 1: Table S1. Overview of Country Programs using Artificial Intelligence to Interpret Chest Radiographs. [file 44263_2023_33_MOESM1_ESM.docx]

**Table S1: Overview of Country Programs using Artificial Intelligence to Interpret Chest Radiographs**

| **Country** | **Program goals** | **CAD used** | **Screening approach** | **Operators and readers** | **Notes/References** |
| --- | --- | --- | --- | --- | --- |
| **Bangladesh** | Initial project goal was to establish linkage between private providers and the TB national program by providing access to quality assured CXR and Xpert testing to patients referred by private providers in Dhaka.  A second implementation project used 14 mobile systems thorugh a collaboration with the NTP to increase TB case detection and use in areas where X-ray facilities are not available. | Primarily CAD4TB and later qXR and Lunit were used for evaluations. | In initial deployment for private sector engagement, people with presumptive TB (adults) were tested by CXR followed by Xpert. CAD read the films for evaluation purposes; it was not used in clinical decision making.  In the second deployment people with presumptive TB and household contacts are tested by CXR and CAD. People with higher score than the nationally adopted threshold value, or those with symptoms are referred for bacteriological testing (Xpert/TruerNAT/Microscopy as available). We expect to share preliminary findings at the union.  Across the two initiatives more than 50,000 people have been screened. | All the images were taken by qualified radiographers in the 3 centers. Expert radiologists re-read all films for a comparison  In second deployment qualified radiographers perform X-rays. A subset of images (higher than threshold score but negative on bacteriological testing) are sent for radiologist interpretation and , reports are shared with the patient. | ^1^,^2^ |
| **India** | Engaging private informal providers for referrals of chest X-ray.  The program compensated for CXR prescribed for TB screening by informal private providers to encourage referrals. 8 private Xray facilities were engaged. | qXR | Private providers verbally screened individuals for TB. Abnormal CAD findings sent to a healthcare worker as text messages generated using the qTrack application, also developed by Qure.ai. All laboratory testing done at designated TB program sites. Approximately 8300 people screened. | CXR conducted in private facilities with radiographers. All CXRs were read by radiologists as well as by the qXR AI software. |  |
| **Malawi** | PROSPECT Study. Individually randomised trial in one urban primary healthcare centre  SCALE study. Community prevalence survey in urban Blantyre, Malawi prior to cluster randomised trial | CAD4TB  qXR | PROSPECT. Adults with cough attending primary care (HIV positive and negative) offered CXR with CAD4TBv5. X-rays done by radiographers. CAD threshold of 45, with confirmation by sputum Xpert.  SCALE. randomly selected participants adults had chest X-ray. All participants with TB symptoms or abnormal CXR submitted two sputum samples  In total around 14,000 people screened | CXR conducted by study radiographer. Digital chest X-rays were additionally read remotely by a consultant radiologist, and participants with abnormal findings identified were recalled by telephone and referred to the clinic or city central hospital with results. | PROSPECT Study^3^  SCALE Study^4^ |
| **Nigeria** | Active case finding in hard to reach rural areas. Closing the gap in TB case detection especially among key populations (nomadic communities and internally displaced persons) who have been shown to have a higher burden of TB in Nigeria | qXR | Symptom and CXR screening read onsite by CAD and people with either CAD-abnormal (.30 or above) or symptoms would be eligible for Xpert testing.  About 10,000 people screened as part of active case finding | A team of 4 people conducted the screening camps including a trained radiographer, screeners, and a site coordinator. An offsite radiologist read abnormal CAD images for potential clinical diagnosis. | ^5^ |
| **Pakistan** | Massive active case finding for TB program with over 50 mobile vans, part of a larger Zero TB cities initiative which began in Karachi- Sindh (province) in 2017, and was later scaled up nationally to four other provinces (KPK, Balochistan, Punjab and Gilgit Baltistan) in the country. The program focused on active screening for TB in a variety of settings such as hospitals, community settings, factories, mines and prisons. | Primarily CAD4TB.  qXR was used during Covid-19 and TB screening | A CAD4TB cut-off score of 70 was used to define presumptive TB; people were then asked to submit a sputum (spot) sample for Xpert MTB/RIF or Xpert Ultra. Those with bacteriologically confirmed test, as well as those that were heavily symptomatic were linked to trained physicians at TB Basic Management Units (BMUs) near their areas of residence.  As part of this case finding program we conducted over 1.5 million chest X-ray screens for TB between 2017-2021.  Covid-19 integrated screening was done an about 46,000 people in 2021. | The x-ray equipment and associated software was handled by trained radiographers who were adept at conducting these x-rays as well as managing the equipment and associated accessories.  TB treating physicians at treatment centers conducted clinical evaluation for clinical diagnosis of Xpert negative presumptive (if required) and initiated treatment. |  |
| **Peru** | Prisons: TB screening program to do active case finding in six women’s prisons  Community based active case finding using mobile vans. | Prisons – qXR  Community-based ACF CAD4TB | Voluntary symptom and screening offered to all women. A digital CXR was obtained for every participant, regardless of symptoms. The findings were analyzed using qXR software and classified as normal or abnormal according to the manufacturer’s default settings (0.5). Sputum samples for Xpert testing were collected from participants who had abnormal CXR, and normal CXR but with a productive cough lasting longer than 14 days or a history of HIV, and had either fever, weight loss, or night sweats.  The mobile units offer free chest radiography to anyone aged ≥4 years, regardless of symptoms. Radiographs is scored by CAD4TB, with a score >50 classified as abnormal. Individuals with abnormal radiographs are given a clinical evaluation by the mobile unit physician who review the chest radiograph obtained during screening. | Women at prisons eligible for Ultra testing were also evaluated by a physician for possible clinical diagnosis of TB and those diagnosed with TB were referred to the local health authority for treatment initiation.  Physician at the mobile unit can determine that there is evidence of TB based on clinical or radiologic criteria and recommend treatment.  A doctor from the TB program at the local health facility confirms the diagnosis and initiates treatment. | ^6^,^7^ |
| **S Africa** | Vukuzazi Community-based multi-disease screening and biobanking study. Residents of the Africa Health Research Institute demographic surveillance area in rural KwaZulu-Natal were screened for multiple conditions (HIV, TB, hypertension, diabetes). TB screening including WHO-4 symptom screen and digital chest x-ray interpreted in the field by CAD. | CAD4TB | In the field, any symptom, a CAD score > 25, or any one able to undergo digital CXR (pregnant woman, infirmity that prevented climbing into the van) triggered sputum collection. Sputum was assessed by Xpert Ultra and MGIT culture.  18,041 adolescents and adult (>15 years of age) were included. | Digital CXRs were also interpreted by an expert radiologist off-site for assessment of normal/abnormal and abnormality suggestive of active TB.  Radiologist-identified abnormality that had been "missed" in the field, triggered attempt at sputum collection. | ^8^ |
| **Vietnam** | Multiple different projects and pilot deployments was used to inform this perspective piece. Large focus on active case finding with mobile vans but also included facility-based screening. | qXR primarily in program deployment.  DrAid (VinBrain) more recently.  Several AI software including Lunit Insight as part of different evaluations. | Positioned the CAD software as a paired read/interpretation with an on-site radiologist at both community screening events and in health facilities. Individuals with abnormal results on either read (even when the CAD software and radiologist findings are discordant) were eligible for follow-on sputum testing with the Xpert MTB/RIF assay.  Close to 20,000 screenings using the AI and human reading in parallel as part of program deployment were conducted. | CXR screening sites were staffed with a radiographer and radiologist as per regulations in Vietnam, who read all images before or in parallel with the AI. AI software was used for EQA after the event when parallel deployment was not possible.  For evaluations of different AI platforms, intermediate and expert radiologists were recruited as reference standards | ^9^,^10^ |
| **Zambia** | This was facility based active case finding project. | CAD4TB | The CAD threshold was set at <=60 Normal. Patients with CAD >=61 received Xpert testing. Presumptive TB was defined as any cough, fever, night sweats or loss of weight among patients presenting to the health facility.  Around 9,400 individuals were screened. | Part of study procedure at health facility with radiographer. Radiologists were not used as part of the evaluations. | First prospective study to evaluate CAD4TB. ZAMPACT Project^11^,^12^ |

**References**

1. Rahman MMTM, Codlin AJ, Rahman MMTM, Nahar A, Reja M, Islam T, et al. An evaluation of automated chest radiography reading software for tuberculosis screening among public- and private-sector patients. Eur Respir J [Internet]. 2017 May 21;49(5):1602159. Available from: http://dx.doi.org/10.1183/13993003.02159-2016

2. Qin ZZ, Barrett R, Ahmed S, Sarker MS, Paul K, Adel ASS, et al. Comparing different versions of computer-aided detection products when reading chest X-rays for tuberculosis. PLOS Digit Heal [Internet]. 2022;1(6):e0000067. Available from: http://dx.doi.org/10.1371/journal.pdig.0000067

3. MacPherson P, Webb EL, Kamchedzera W, Joekes E, Mjoli G, Lalloo DG, et al. Computer-aided X-ray screening for tuberculosis and HIV testing among adults with cough in Malawi (the PROSPECT study): A randomised trial and cost-effectiveness analysis. Barnabas R V., editor. PLOS Med [Internet]. 2021 Sep 9;18(9):e1003752. Available from: http://dx.doi.org/10.1371/journal.pmed.1003752

4. Feasey HRA, Khundi M, Nzawa Soko R, Nightingale E, Burke RM, Henrion MYR, et al. Prevalence of bacteriologically-confirmed pulmonary tuberculosis in urban Blantyre, Malawi 2019–20: Substantial decline compared to 2013–14 national survey. PLOS Glob Public Heal [Internet]. 2023;3(10):e0001911. Available from: http://dx.doi.org/10.1371/journal.pgph.0001911

5. John S, Abdulkarim S, Usman S, Rahman MT, Creswell J. Comparing tuberculosis symptom screening to chest X-ray with artificial intelligence in an active case finding campaign in Northeast Nigeria. BMC Glob Public Heal [Internet]. 2023 Oct 6;1(1):17. Available from: https://doi.org/10.1186/s44263-023-00017-2

6. Chadha VK, Praseeja P. Active tuberculosis case finding in India – The way forward. Indian J Tuberc [Internet]. 2019;66(1):170–7. Available from: https://doi.org/10.1016/j.ijtb.2018.05.014

7. Puma D, Geadas C, Calderon RI, Yuen CM, Jiménez J, Córdova M, et al. Active case-finding for TB among incarcerated women in Peru. Int J Tuberc Lung Dis. 2023;27(10):784–6.

8. Gunda R, Koole O, Gareta D, Olivier S, Surujdeen A, Smit T, et al. Cohort Profile: The Vukuzazi (‘Wake Up and Know Yourself’ in isiZulu) population science programme. Int J Epidemiol [Internet]. 2022 Jun 13;51(3):e131–42. Available from: https://academic.oup.com/ije/article/51/3/e131/6446138

9. Codlin AJ, Dao TP, Vo LNQ, Forse RJ, Van Truong V, Dang HM, et al. Independent evaluation of 12 artificial intelligence solutions for the detection of tuberculosis. Sci Rep [Internet]. 2021 Dec 13;11(1):23895. Available from: https://doi.org/10.1038/s41598-021-03265-0

10. Vo LNQ, Codlin A, Ngo TD, Dao TP, Dong TTT, Mo HTL, et al. Early Evaluation of an Ultra-Portable X-ray System for Tuberculosis Active Case Finding. Trop Med Infect Dis [Internet]. 2021 Sep 4;6(3):163. Available from: https://www.mdpi.com/2414-6366/6/3/163

11. Muyoyeta M, Moyo M, Kasese N, Ndhlovu M, Milimo D, Mwanza W, et al. Implementation research to inform the use of xpert MTB/RIF in primary health care facilities in high TB and HIV settings in resource constrained settings. PLoS One. 2015;10(6):1–18.

12. Muyoyeta M, Kasese NC, Milimo D, Mushanga I, Ndhlovu M, Kapata N, et al. Digital CXR with computer aided diagnosis versus symptom screen to define presumptive tuberculosis among household contacts and impact on tuberculosis diagnosis. BMC Infect Dis. 2017;17(1):1–8.
